# Revealing RCOR2 as a regulatory component of nuclear speckles

**DOI:** 10.1186/s13072-021-00425-4

**Published:** 2021-11-24

**Authors:** Carlos Rivera, Daniel Verbel-Vergara, Duxan Arancibia, Anna Lappala, Marcela González, Fabián Guzmán, Gianluca Merello, Jeannie T. Lee, María Estela Andrés

**Affiliations:** 1 Department of Cellular and Molecular Biology, Faculty of Biological Sciences, Pontificia Universidad Católica de Chile, Avenida Del Libertador Bernardo O’Higgins 340, 8320000 Santiago, Chile; 2 Department of Molecular Biology, Massachusetts General Hospital, 185 Cambridge Street, CPZN 6624, Boston, MA 02114 USA; 3 Department of Genetics, The Blavatnik Institute, Harvard Medical School, Boston, MA 02114 USA

**Keywords:** CoREST, CoREST2, RCOR2, Nuclear speckles, Membrane-less organelles, SRRM2, SRSF7, SC35, SRSF2, SON

## Abstract

**Background:**

Nuclear processes such as transcription and RNA maturation can be impacted by subnuclear compartmentalization in condensates and nuclear bodies. Here, we characterize the nature of nuclear granules formed by REST corepressor 2 (RCOR2), a nuclear protein essential for pluripotency maintenance and central nervous system development.

**Results:**

Using biochemical approaches and high-resolution microscopy, we reveal that RCOR2 is localized in nuclear speckles across multiple cell types, including neurons in the brain. RCOR2 forms complexes with nuclear speckle components such as SON, SRSF7, and SRRM2. When cells are exposed to chemical stress, RCOR2 behaves as a core component of the nuclear speckle and is stabilized by RNA. In turn, nuclear speckle morphology appears to depend on RCOR2. Specifically, RCOR2 knockdown results larger nuclear speckles, whereas overexpressing RCOR2 leads to smaller and rounder nuclear speckles.

**Conclusion:**

Our study suggests that RCOR2 is a regulatory component of the nuclear speckle bodies, setting this co-repressor protein as a factor that controls nuclear speckles behavior.

**Supplementary Information:**

The online version contains supplementary material available at 10.1186/s13072-021-00425-4.

## Introduction

Eukaryotic gene expression requires the coordination of macromolecular complexes to ensure the appropriate synthesis and processing of RNAs. Coactivator and corepressor complexes are recruited into chromatin and modulate gene expression by inducing covalent histone modifications and physical displacement of nucleosomes, causing a local change in chromatin accessibility [1]. The RCOR (CoREST) family of transcriptional corepressors has been characterized based on its ability to silence neuronal genes in non-neuronal cells and during early stages of neuronal differentiation [2]. RCOR corepressors behave as molecular bridges that bring enzymatic activities to remove transcriptionally permissive histone modifications.

The most stable interactions are established with the H3K4me1/2-demethylase LSD1 (KDM1A), and the histone deacetylases HDAC1 and HDAC2 [3]. Intriguingly, RCOR2 (CoREST2) and RCOR3 (CoREST3) are weaker repressors than RCOR1 (CoREST, CoREST1) [4], and this feature might be related to their effects on the coordination of LSD1 and HDAC1/2 activities [4, 5]. Accordingly, RCOR1 can efficiently stimulate LSD1-mediated H3K4me1/2 demethylation on nucleosomal substrates, while RCOR2 only exerts a subtle LSD1 stimulation [6, 7]. This illustrates the existence of differential biochemical properties among RCOR family members. Interestingly, RCOR2-mediated repression does not require HDAC1/2 activity [4]. In addition, in vivo evidence has attributed specific functions to RCOR2 in developmental regulation, as it is the only member of the RCOR family which is important for the maintenance of stem cell state across the cell cycle and is required to achieve pluripotency when reprogramming fibroblasts [8]. RCOR2 has also been reported to regulate brain cortex development in mice [9, 10].

Apart from the unique properties already reported for RCOR2 and its biological impact, much remains unclear about how RCOR2 regulates chromatin and represses gene expression. Because specific immunostaining of RCOR2 shows a punctate nuclear distribution in interphasic cells, which significantly differs from other RCOR proteins [10, 11], we hypothesize that biological compartmentalization may be involved in the regulation of its particular functions. In this report, we show through high-resolution microscopy and biochemical approaches that RCOR2 is constitutively recruited to interchromatin granule clusters (IGCs) known as "nuclear speckles". These nuclear bodies are membrane-less organelles formed by assemblies of 20-nm ribonucleoprotein particles connected by narrow fibers [12] constituting a phase-separated granule that behaves as nucleoplasmic liquid droplets [13]. We describe RCOR2 as a core component that actively regulates nuclear speckle morphology and thereby expand the canonical roles associated with RCOR2.

## Results

### RCOR2 is compartmentalized at nuclear speckles

While examining published RCOR2 immunofluorescence studies performed on murine brain slices or neuronal primary cultures, we noticed an intriguing subcellular distribution of RCOR2 showing a nuclear punctate pattern [10, 11]. We wondered whether this nuclear distribution could be detected in different cell lines. After testing different antibodies, we selected a Prestige-validated, Protein-Atlas recommended, anti-RCOR2 antibody (Millipore-Sigma, #HPA021638) since when validating its specificity, it preferentially detected a band with the predicted RCOR2 molecular weight (Additional file 1: Fig. S1A) and recognized overexpressed Myc-tagged RCOR2 both by Western blot (Additional file 1: Fig. S1B) and immunofluorescence (Additional file 1: Fig. S1C). In addition, its immunofluorescent signal disappeared when pre-incubated with its peptide antigen, but not with a non-specific histone H3 peptide (Additional file 1: Fig. S1D). Furthermore, it detected the loss of endogenous RCOR2 when performing knock down experiments both by Western blot and immunofluorescence (Additional file 1: Fig. S1E and F).

**Fig. 1:**
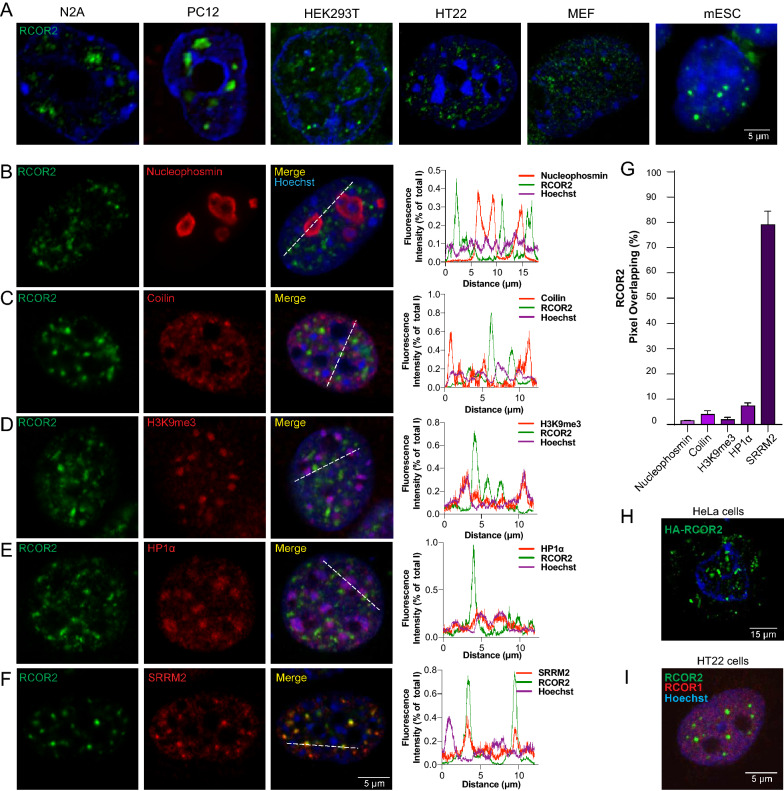
RCOR2 colocalizes with nuclear speckles. **A** Different cell types immunolabeled for RCOR2 (green). **B**–**F** HT22 cells were stained with double immunolabeling of RCOR2 (green) and nucleophosmin (**A**), coilin (**B**), H3K9me3 (**C**), HP1α (**D**) or SRRM2 (**E**) as molecular markers of nuclear bodies or chromatin condensates (red). For each staining procedure, the spatial correlation of both fluorophores plus Hoechst staining was tracked according to the white dashed lines. Right-side plots show their normalized fluorescence intensity plotted against the position on the dashed line. Images are representative of three independent stainings. **G** RCOR2 overlapping degree is expressed as the percentage of thresholded RCOR2 pixels overlapping territories of red channel pixels. Percentages were calculated based on RCOR2 thresholded Manders’ coefficients analyzed by JACoP ImageJ-plugin on independent stainings. **H** HeLa cells were transfected with a plasmid to over-express recombinant HA-RCOR2. Cells were immunostained with an anti-HA epitope antibody (green). **I** Double immunolabeling of RCOR1 (red) and RCOR2 (green) on HT22 cells.

We therefore carried out RCOR2 immunostaining on PC-12, N2A, HEK293T, HT22, MEF and mESC cells. In all cases, a nuclear enrichment was observed with different ratios of clustered and dispersed nucleoplasmic patterns (Fig. 1A), confirming that RCOR2 is distributed in the nucleus as puncta with differences in size and abundance depending on the cell type. This observation raised the possibility that RCOR2 may be recruited to some type of nuclear body or chromatin condensate. To test this, we carried out double immunofluorescence labeling of RCOR2 and protein markers for nucleoli (nucleophosmin), Cajal bodies (coilin), pericentric heterochromatin (Heterochromatin protein 1α, HP1α; and H3K9me3). To label nuclear speckles, we used a monoclonal antibody originally described and widely used to recognize the SC35/SRSF2 protein. However, it was recently shown that the actual target of this antibody corresponds to the serine arginine repetitive matrix protein 2 (SRRM2) and it can also detect serine and arginine splicing factor 7 (SRSF7), two bona fide nuclear speckle residents [14].

As expected, RCOR2 showed an intranuclear punctate distribution, enriched at the interchromatin space since it was excluded from regions with intense Hoechst staining in several cell types (Fig. 1A, B). RCOR2 puncta were excluded from nuclear territories occupied by nucleoli, Cajal bodies, and chromocenters (pericentric heterochromatin), as showed by nucleophosmin, coilin, and H3K9me3/HP1α co-staining, respectively (Fig. 1B–E). Quantitation of RCOR2 colocalization with these nuclear bodies showed less than 8.5% of its fluorescence signal overlapping them (Fig. 1G), and no correlation between their fluorescence intensity profiles (Fig. 1B–E). Conversely, when we analyzed the co-staining between RCOR2 and nuclear speckles by SRRM2 immunofluorescence, we found a strong correlation of their intensities and about 74% of RCOR2 signals overlapping SRRM2 territories in HT22 cells (Fig. 1F, G). We also observed that transiently expressed HA-RCOR2 mimicked the punctate pattern in HeLa cells (Fig. 1H). Furthermore, this localization was specific for this member of the RCOR family, as RCOR1 did not exhibit this pattern (Fig. 1I). Overall, our data showed that a large fraction of RCOR2 localizes to nuclear speckles.

### RCOR2 is recruited to nuclear speckles in the mouse brain

**Fig. 2:**
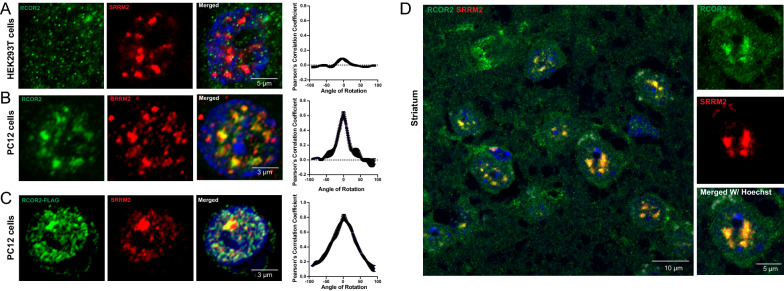
RCOR2 is recruited to nuclear speckles in the mouse brain. **A**, **B** Comparison of colocalization between RCOR2 (green) and SRRM2 (red) in HEK293T (**A**) and PC12 (**B**) cells. Right-side plots show Van Steensel’s colocalization test profiles. **C** Colocalization analysis between overexpressed FLAG-tagged RCOR2 (green) and SRRM2 (red) in PC12 cells. Right-side plot shows Van Steensel’s colocalization test. **D** Tissue immunofluorescence of SRRM2 (red) and RCOR2 (green) in mice striatum slices. The right panels show a zoomed-in nucleus, illustrating speckles found in single nuclei of the brain tissues

We wondered next whether RCOR2 recruitment to nuclear speckles could be a broader phenomenon. We analyzed the RCOR2/SRRM2 colocalization in different cell types. While nuclear speckle particles looked similar both in HEK293T cells and PC12 cells, the former depicted RCOR2 puncta whose particles were smaller and more disperse than in PC12 cells (Fig. 2A, B). Notably, RCOR2 recruitment to nuclear speckles was much stronger in PC12 than in HEK293T cells, since colocalization with SRRM2 was only partial on the latter, suggesting its recruitment is a cell type dependent process. Nevertheless, RCOR2-FLAG overexpression on PC12 cells imitated the endogenous RCOR2 distribution whose puncta colocalized with SRRM2 (Fig. 2C). This colocalization of RCOR2 and SRRM2 was also observed in mouse brain, as detected in mouse striatum tissue slices (Fig. 2D and Additional file 5: Video S1). Similar results were observed in prefrontal-cortex slices (Additional file 2: Fig. S2A).

### RCOR2 localizes to the core of nuclear speckles

**Fig. 3:**
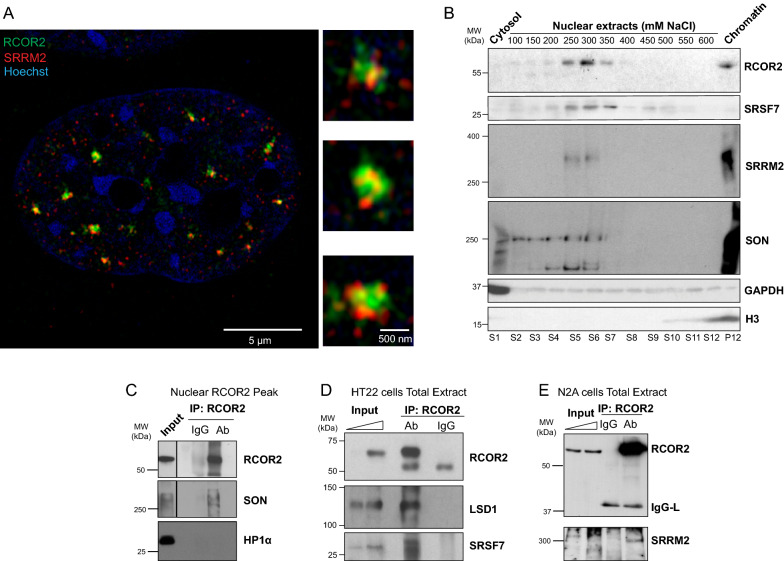
RCOR2 associates with nuclear speckles at their core region. **A** RCOR2 (green) and SRRM2 (red) immunolabeling in HT22 cells visualized at super-resolution confocal acquisition. Hoechst is visualized in blue color. Right panels show zoomed-in individual nuclear speckles. **B** Western blot analysis of RCOR2, SRRM2, SON, and SRSF7 distribution on cytosolic, nuclear and chromatin fractions of HT22 cells. GAPDH and H3 were assayed as cytosolic and chromatin markers, respectively. Western blot is representative of two independent experiments. Numbers in nuclear fractions indicate the NaCl concentration (mM) at which the soluble fraction was extracted. S: supernatant. P: pellet. **C** Western blot analysis of co-immunoprecipitation of RCOR2, SON and HP1α in nuclear soluble fractions enriched in RCOR2 from HT22 fractionation experiments. Ab: antibody used to immunoprecipitate RCOR2. IgG: immunoglobulin G used as a specificity control. **D**, **E** Western blot analysis of co-immunoprecipitation of RCOR2 and nuclear speckle components using HT22 (D) and N2A (E) total cell extracts as input. LSD1 was assayed as a positive control for RCOR2 interactors. Ab: Antibody used for immunoprecipitation of RCOR2. IgG: Immunoglobulin G used as a specificity control

Nuclear speckles display a structure defined by an inner core where SRRM2 and SON locate, and this center is coated by a second layer containing RNAs such as Malat1 and U1 whose periphery is surrounded by poly-(A)-RNAs and other factors [15]. This feature inspired us to determine at which region of the nuclear speckle architecture RCOR2 is recruited. To this end, we examined the colocalization between RCOR2 and SRRM2 at high resolution by Airyscan confocal microscopy with a 2D super-resolution acquisition mode, which can achieve ~ 120 nm resolution in XY planes [16]. We detected a strong colocalization between both marks (Fig. 3A), indicating that RCOR2 recruitment occurs at the core region of nuclear speckles.

Previous studies have shown that nuclear speckle components can be co-extracted from nuclear fractions upon fractionating them with hypertonic buffers [17]. Thereby, we hypothesized that if RCOR2 is actually interacting with nuclear speckle core-components, we could detect their co-extraction and interactions. Thus, we first separated cytosol and nuclei from HT22 cells, and then sequentially extracted nuclear fractions by incubating the nuclei with increasing salt concentrations steps (Additional file 2: Fig. S2B). We found RCOR2 distributed in two main subnuclear fractions, the first one was extracted between 250 and 350 mM NaCl and the second one resisted all the salt-induced extractions, remaining enriched in the chromatin pellet (Fig. 3B). Supporting the idea that RCOR2 belongs to the nuclear speckles, we observed that SRRM2, SON and SRSF7 were co-extracted with RCOR2 at fractions between 250 and 350 mM NaCl (Fig. 3B). In addition, we performed co-immunoprecipitation experiments starting from the nuclear RCOR2 peak and, in conditions where HP1ɑ, which does not interact with RCOR2, was absent; we were able to detect SON as part of the RCOR2 immunocomplex, suggesting RCOR2 forms complexes with a speckle core component in the extracted fractions (Fig. 3C). Finally, we decided to test if RCOR2 interactions with nuclear speckle components could also be detected when performing co-immunoprecipitation assays starting from HT22 and N2A whole-cell protein extracts. In this way, RCOR2 immunoprecipitation effectively precipitated its bona fide interactor LSD1 and SRSF7 in HT22 cells (Fig. 3D) and HEK293T cells (Additional file 2: Fig. S2C), while it co-precipitated SRRM2 in N2A cells (Fig. 3E). Altogether these data suggest that the RCOR2 is a core component of nuclear speckles.

### RCOR2 associates with nuclear speckles inside its core region stabilized by an RNA component

**Fig. 4:**
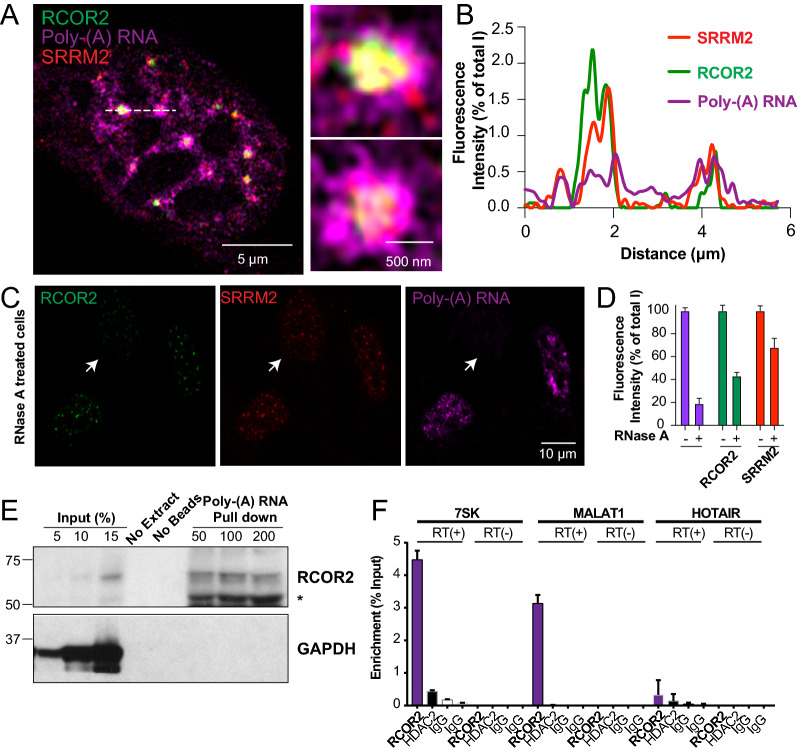
An RNA component stabilizes RCOR2 at nuclear speckles. **A** Merged representation of RCOR2 (green), SRRM2 (red), and Poly(A)-RNA (magenta) triple immunostaining at super-resolution confocal acquisition in HT22 cells. Right panels show zoomed-in individual nuclear speckles. **B** Fluorescence correlation plot showing the normalized fluorescence intensity of each mark plotted against the position on the dashed line. **C** RCOR2 (green), SRRM2 (red), and poly(A)-RNA (magenta) labeling of RNAse A treated HT22 cells visualized at regular confocal acquisition. Arrows indicate a representative cell that lost its RNA content after RNAse A treatment. **D** Quantitation of normalized cell fluorescence intensity for each channel of cells mock-treated and RNAse A-treated. Quantitations included 20 cells from 3 biological replicates. **E** Western blot analysis of RCOR2 and GAPDH to test their coprecipitation after poly(A)-pull-down. Increasing concentrations of protein input from HT22 cells were loaded to estimate enrichment after pull down. No loaded extract and extract without beads were subjected to the same procedure as specificity controls. **F** Real-time quantitative PCR results showing 7SK and MALAT1 fragment amplification after performing RNA immunoprecipitation on HT22 nuclear extracts with an anti-RCOR2 antibody. IgG was used as specificity control and qPCR without reverse transcription (RT(-)) was analyzed to discard eventual DNA concentration after immunoprecipitations. HDAC2 antibody was used as a negative control and the nuclear lncRNA HOTAIR was analyzed as a negative control. Results are representative of two different biological replicates

As recently shown, SRRM2 forms the core region of nuclear speckles [14], while both a subset of polyadenylated pre-mRNAs and exon-junction processing complexes are enriched at the periphery [18, 19]. To further inquire about the position of RCOR2 in the nuclear speckles, we established an immuno-RNA FISH protocol that enabled us to perform a triple fluorescence labeling of RCOR2, SRRM2 and poly-adenylated RNA (poly(A)-RNA). Previous studies suggested that poly(A)-RNAs are distributed between nuclear speckles and the nucleoplasmic space [22]. Super-resolution acquisition mode of Airyscan confocal microscopy enabled us to observe that RCOR2, SRRM2, and poly(A)-RNAs colocalized at nuclear speckles, with poly(A)-RNAs decorating their periphery, and forming a fiber-like structures, which seemed to connect different speckles (Fig. 4A). Both RCOR2 and SRRM2 appeared to be in close proximity with poly(A)-RNA, as no space was observed between them (Fig. 4A, B).

Considering the sub-compartmentalized layers of nuclear speckles [15], some components, such as the stress response protein Gadd45 are recruited in an RNA-dependent manner [20], while others, such as PRPF40B are not [21]. To test whether RNA molecules stabilize RCOR2 at nuclear speckles, we analyzed RCOR2 localization after partial digestion of RNA. Here, we pre-treated permeabilized cells with DNAse-free RNAse A before fixation and then carried out triple labeling of RCOR2, SRRM2, and poly(A) RNAs to correlate RCOR2 levels with remaining RNA content in cells of the same samples. Under control conditions, the permeabilization and subsequent mock treatment did not change the subnuclear distribution of RCOR2 and SRRM2 (Additional file 2: Fig. S2D). However, under RNAse A treatment, cells that lost more than 80% of their poly(A)-RNAs showed around 60% decrease RCOR2 fluorescence intensity (Fig. 4C and D). Also, SRRM2 fluorescence intensity dropped around 40%, indicating a partial RNA-dependence on its nuclear speckle localization when analyzing it with in situ internal RNA degradation controls. These data suggest that RCOR2 localization within nuclear speckles depends on RNA or is stabilized by RNA.

Accordingly, we tested if RCOR2 is an RNA-binding protein by subjecting native, chromatin-free cell extracts of HT22 cells to poly(A)-RNA pull-down using oligo-dT-conjugated beads. In conditions where GAPDH, that does not bind RNA, was not pulled down, we detected strong enrichment of RCOR2 in the beads, pulling down more than 30% of the input protein (Fig. 4E), indicating that RCOR2 binds poly(A)-RNA in cells. Finally, since we observed close proximity and even colocalization between RNA and RCOR2, we tested if RCOR2 can interact with non-coding RNAs that are enriched in the second layer of nuclear speckles by native RNA immunoprecipitation (RIP) assays. In conditions where no DNA was amplified by qPCR, we detected specific interactions between RCOR2 and the speckle RNAs MALAT1 and 7SK (Fig. 4F). To further confirm the specificity of this interaction, we compared our results with HDAC2 RIP as another nuclear protein whose interaction with RCOR2 is neglectable [4]. As shown in Fig. 4F, none of the tested RNAs was pulled down with anti-HDAC2 antibody. In addition, HOTAIR, another nuclear LncRNA, was not enriched by RCOR2 antibodies (Fig. 4F). Therefore, RCOR2 specifically interacts with nuclear speckle RNAs from their second layer and its localization is impaired after in situ RNA degradation.

### RCOR2 behaves as a core component of nuclear speckle

**Fig. 5:**
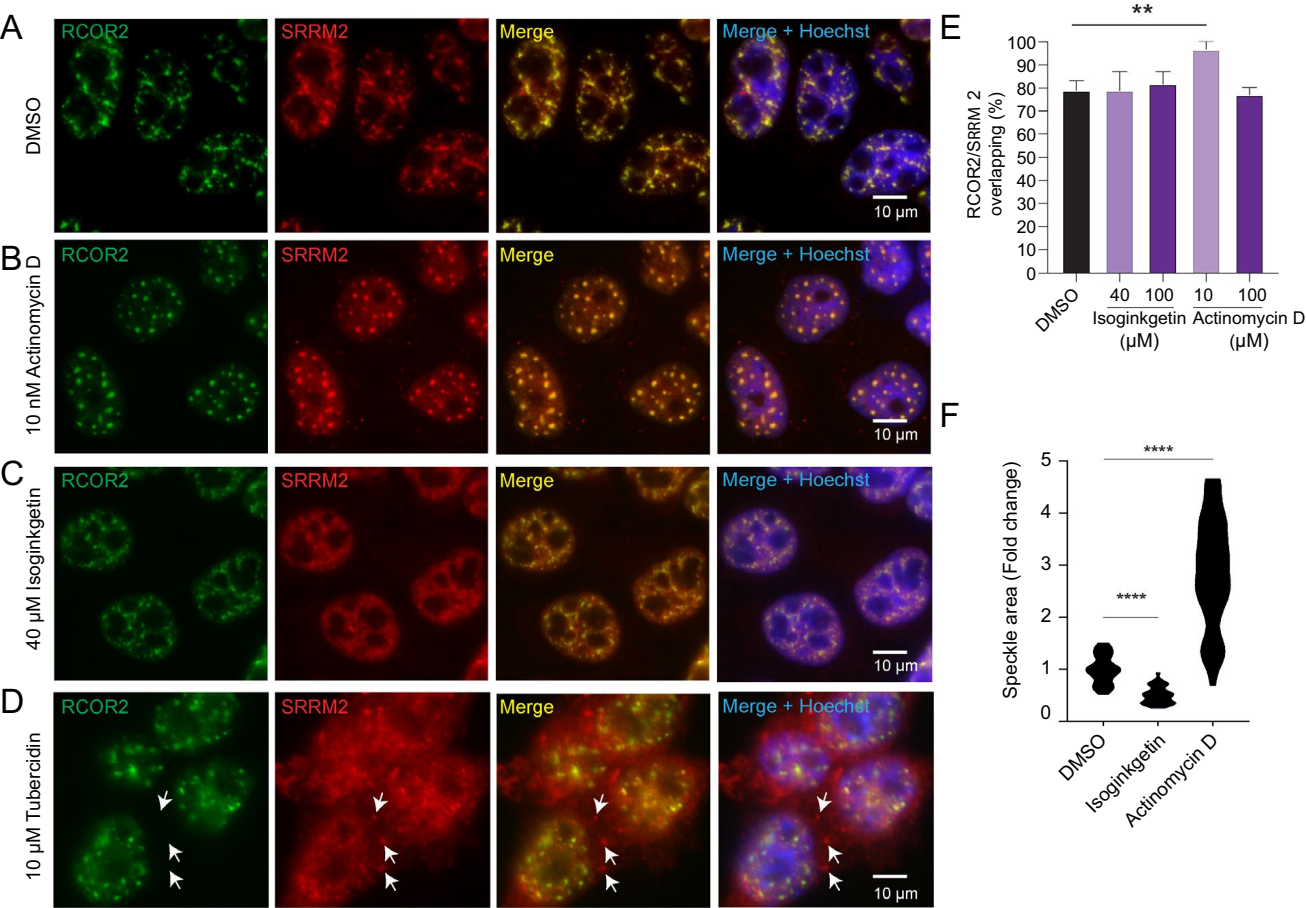
RCOR2 behaves as a core component of nuclear speckles. **A**–**D** HeLa cells were stained against RCOR2 (green) and SRRM2 (red) after treatments with DMSO vehicle (**A**), actinomycin D (**B**), isoginkgetin (**C**) and tubercidin (**D**). Arrows indicate cytoplasmic SRRM2 aggregates. Images are representative of two independent experiments. **E** RCOR2 overlapping degree is expressed as the percentage of thresholded RCOR2 pixels overlapping SRRM2 territories. Percentages were calculated based on RCOR2 thresholded Manders coefficients analyzed by JACoP ImageJ-plugin on independent stainings. **p < 0.01. **F** Speckle area quantitation under isoginkgetin and actinomycin D treatments. The area was measured in 10 cells measuring areas of 10 speckles per cell on each condition. Results were normalized against DMSO control values. Unpaired t-tests were performed for each condition against mock-treated cells to estimate statistical significance. ****p < 0.001

The morphology of nuclear speckles changes significantly with the inhibition of transcription [22]. Consistently, variations in nuclear speckle sizes result from speckle fusion and over-recruitment of nucleoplasmic factors. Our previous data showing that RCOR2 localizes in the nuclear speckle core prompted us to test if it could react to this cellular stress as other core components do. To this end, we treated HeLa cells with actinomycin D, which blocks transcriptional elongation. As expected, nuclear speckles became bigger and rounder (Fig. 5A, B, F and Additional file 3: Fig. S3A). RCOR2 remained recruited to these bodies, as observed by its colocalization with SRRM2 (Fig. 5B, E). Thus, RCOR2 responded as a core nuclear speckle component when exposed to this treatment.

We also tested the effect of isoginkgetin, a biflavonoid that inhibits the recruitment of the U4/U6/U5 snRNP complex into the spliceosome, by blocking its assembly at an early stage of the splicing cycle [23]. The exposure of cells to low and high concentrations of isoginkgetin caused a decrease in nuclear speckle sizes (Fig. 5C, F and Additional file 3: Fig. S3B). Meanwhile, the signal for RCOR2 remained similar to controls and colocalizing with SRRM2 (Fig. 5C, E and Additional file 3: Fig. S3B). This data suggests that RCOR2 behaves as a core component of nuclear speckles when cells are exposed to chemicals that inhibit spliceosome assembly. We finally tested tubercidin to induce disassembly of the nuclear speckles. This adenosine analog was can displace poly(A)-RNA from the nucleus causing release of some splicing factors from nuclear speckles, stress granule formation and mRNA export defects [24, 25]. In our model, tubercidin caused a partial dispersion of SRRM2 to the nucleoplasm and cytoplasm, with some recruitment of this splicing factor to cytoplasmic granules (Fig. 5D). Surprisingly, RCOR2 maintained a clustered distribution, being still colocalizing with nuclear SRRM2. Altogether, these data indicate that RCOR2 behaves as a nuclear speckle core component even under stress conditions that challenge speckle morphology and integrity.

### RCOR2 stabilizes the morphology of nuclear speckles

**Fig. 6:**
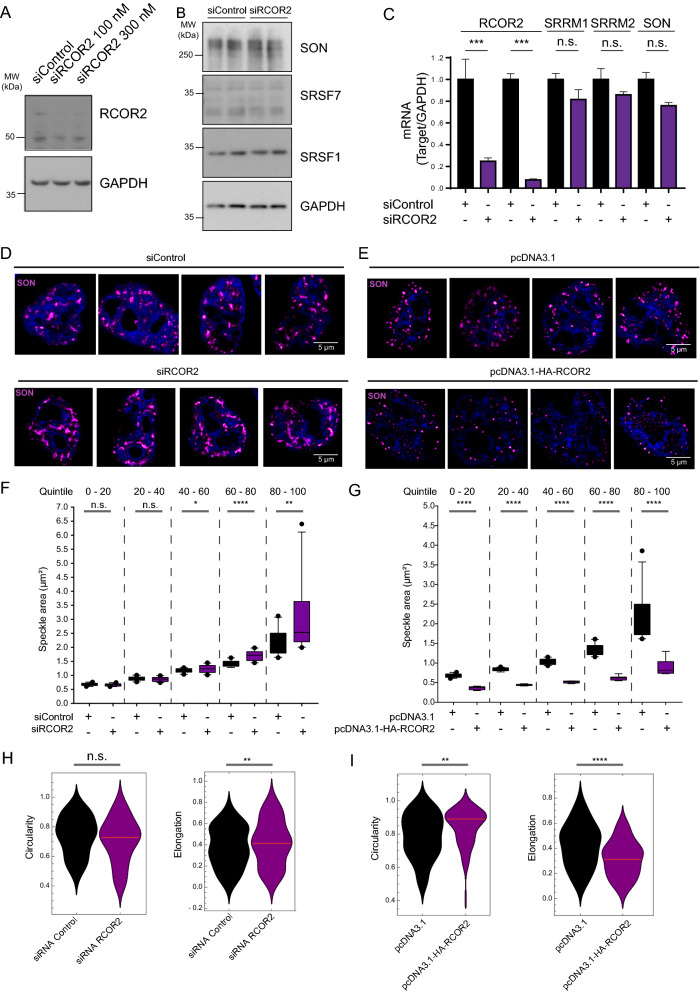
RCOR2 regulates nuclear speckle morphology. **A** Western blot analysis of RCOR2 levels under RCOR2 knockdown in HeLa cells. GAPDH was assayed as a loading control. Panel is representative of three biological replicates. **B** Western blot analyses of nuclear speckle components after knocking RCOR2 down. GAPDH was assayed as a loading control. The blot includes two biological replicates per condition. **C** RT-qPCR results normalized to GAPDH as a constitutively expressed transcript in control and siRCOR2 cells. RCOR2 mRNA was analyzed with two different sets of primers targeting different RCOR2 exons. Results are representative of three biological replicates. ***p < 0.01. **D** SON immunostaining (magenta-hot) on HeLa cells transfected with siControl or siRCOR2 siRNAs. **E** SON immunostaining (magenta-hot) on HeLa cells transfected with pcDNA3.1 or pcDNA3.1-HA-RCOR2 plasmids. **F**–**G** Speckle area was measured after segmenting SON immunostainings from knockdown (**F**) and overexpression (**G**) experiments. Particle area was measured and the population values were grouped into quintiles. Each quintile was compared to its respective control group and statistical significance was tested. *p < 0.05. **p < 0.01. ****p < 0.001. **H** Circularity and elongation of speckles were measured on RCOR2 knockdown samples. Statistical significance was tested on the top 50% particles from each condition. Red lines represent median values. n.s: non significant. **p < 0.01. **I** Circularity and elongation of speckles were measured on RCOR2 overexpression samples. Statistical significance was tested on the whole population of particles from each condition. Red lines represent median values. **p < 0.02. ****p < 0.001

To determine if RCOR2 localization within the nuclear speckle core is of functional significance, we perturbed RCOR2 levels inside cells. First, we knocked down RCOR2 with siRNA in HeLa cells and achieved a significant decrease in RCOR2 mRNA and protein levels without affecting other nuclear speckle components (Fig. 6A–C). The latter finding indicates that RCOR2 is not regulating cellular levels of the core components. We then asked if RCOR2 could affect the morphology of nuclear speckles by analyzing the staining pattern of their core component SON. Indeed, cells lacking RCOR2 depicted bigger nuclear speckles compared to control condition (Fig. 6D). Second, we transiently overexpressed RCOR2 in HeLa cells and found smaller nuclear speckles compared to the control condition (Fig. 6E).

To confirm these observations, we analyzed the area of SON particles under each condition after segmenting the images to identify single particles from each sample (Additional file 4: Fig. S4). We sorted the distribution of the population area values dividing it into quintiles and this allowed us to detect that RCOR2 knockdown led to a significant increase in the area of nuclear speckles coming from quintiles comprising the bigger speckles from each sample (Fig. 6F). Accordingly, we observed that RCOR2 overexpression led to a significant decrease on size of speckles from all the quintiles analyzed. In parallel, we defined two functions to complement our analyses: speckle-elongation (1 – width/height) and speckle-circularity (2•π•equivalent disk radius/polygonal length) that allowed us to study their changes when modulating RCOR2 levels in HeLa cells. RCOR2 knock down produced a significant increase in nuclear speckle elongation when analyzing the Top 50% values from each condition, yet no significant changes were observed when comparing circularity (Fig. 6H). Consistently, RCOR2 overexpression significantly produced rounder and less-elongated nuclear speckles (Fig. 6I). These observations together suggest that RCOR2 levels regulate the size and morphology of nuclear speckles.

## Discussion

In this work, we report by different approaches and in different cell types that RCOR2 is a core component of nuclear speckles that regulates their morphology. These findings open a new layer of biological phenomena linked to RCOR2-specific functions on the regulation of gene expression, beyond its canonical role as a transcriptional corepressor. Although some proteins that behave as transcriptional corepressors, such as the methyl-CpG binding protein 2 (MeCP2) and nuclear receptor corepressor 2 (N-CoR2) [26, 27], have been identified as components of nuclear speckles [26], their role in these nuclear bodies is unknown. To our knowledge, along with a recent report showing p53 cross-talking with nuclear speckles [28] this is one of the first characterizations of a transcriptional regulator exerting a non-canonical function on the homeostasis of nuclear speckles.

### Sub-nuclear distribution of RCOR2 reflects a novel non-canonical function

Classical reports suggest that many nuclear speckle-associated factors, especially the ones involved in pre-mRNA processing, are dynamically distributed between speckles and the nucleoplasm. They propose the nucleoplasmic pool as a subpopulation available to act directly in pre-mRNA processing events while transcription is occurring, proposing nuclear speckles as their storage site [29, 30]. This idea has been supported by live-cell fluorescence microscopy data showing an increase in the speed of pre-mRNA processing when nuclear speckles are disassembled, which correlates with an increase in nuclear speckle factors available in the nucleoplasm [31]. Considering its specific biochemical features and functions among RCOR proteins, RCOR2 is understood as a member that evolved particular functions [4, 8, 10]. This report adds novel specific features to this protein. We showed in different biological systems, ranging from cell lines to murine tissues, that RCOR2 is concentrated inside the nucleus and highly enriched in nuclear speckles. This feature seems to be specific for this RCOR-family member since the immunostaining of RCOR1 do not show similar punctate patterns [11]. Accordingly, our data showed that RCOR1 is segregated from RCOR2 puncta, suggesting it is not present at nuclear speckle territories.

Although additional studies are needed to decode the steady-state distribution of RCOR2 between nuclear speckles, nucleoplasm and chromatin, our microscopy and biochemical data showed RCOR2 distributed among nuclear speckles and chromatin. Since RCOR2 has been genomically found on active chromatin regions marked by H3K4me1 [10], it would be interesting to study if there is a shuttling mechanism where nuclear RCOR2 could be dynamically transitioning between nuclear speckles and active chromatin regions, which have been significantly found in close proximity to these nuclear bodies [32].

### An RNA component allows for the localization of RCOR2 at the core of nuclear speckles

We showed by high-resolution microscopy that RCOR2 localizes at the center of nuclear speckles in close apposition with SRRM2, being surrounded by poly(A)-RNAs that decorate their periphery. Using whole cell protein extracts, we were able to detect RCOR2 in the same immunocomplexes with core speckle components, and between RCOR2 and the speckle associated non-coding RNAs MALAT1 and 7SK, confirming that RCOR2 is a stable component of nuclear speckles in different cell lines. Also, we confirmed the interaction between RCOR2 and SON using a soluble nuclear fraction that co-extracted RCOR2 and nuclear speckle components. In addition, we found a strong enrichment of RCOR2 at fractions where poly(A)-RNAs were precipitated, which correlate with our microscopy findings since we could not detect physical separation between the RCOR2 and poly(A)-RNAs even at high resolution, motivating us to propose RCOR2 as an RNA binding protein. RNA-binding properties of RCOR2 must be important for its nuclear speckle localization, given that RNAse A treatment significantly reduced the levels of the protein inside the nucleus. This supports the idea that RNAs stabilize RCOR2 inside nuclear speckles. Further studies will focus on which RNAs are the strongest binding partners of RCOR2 and how their interactions may impact the RCOR2 function.

### RCOR2 behaves as a core component of nuclear speckles

Because we found RCOR2 at the core of nuclear speckles, we tested if it could behave as a bona-fide core component. As an initial approach, we inhibited transcription with actinomycin D, which blocks transcription at elongation [33]. Transcription inhibitors induce larger and rounder shapes in nuclear speckles [34]. In this context, we observed that under actinomycin D treatment, RCOR2 colocalization with SRRM2 was significantly increased, supporting that RCOR2 distribution is displaced towards nuclear speckles when transcription is inhibited. Additionally, this evidence suggests that some fraction of RCOR2 may be recruited back to nuclear speckles when transcription elongation is blocked. Given that under transcriptional inhibition RCOR2 behaved as SRRM2 but not as MALAT1, which is not a core component and is relocated to the nucleoplasm under this treatment [35], our data support RCOR2 as core component of nuclear speckles.

On the other hand, Isoginkgetin-induced inhibition of spliceosome assembly [23] did not disrupt RCOR2 and SRRM2 colocalization, even when a 60% reduction of speckle’s size was observed. As RCOR2 localization at nuclear speckles was not affected, we could disregard spliceosome assembly as a driver of the recruitment of RCOR2 on nuclear speckles. Finally, given that nuclear speckles disassembly using tubercidin did not modify RCOR2 colocalization with SRRM2, our data evidenced a core-component behavior of RCOR2.

### RCOR2 regulates the morphology of nuclear speckles

Our functional perturbation experiments depicted RCOR2 as a player on the regulation of nuclear speckles morphology. RCOR2-overexpressing cells showed smaller and more circular speckles, suggesting RCOR2 as a regulator of nuclear speckle morphology. In accordance with this, the lack of RCOR2 resulted in cells harboring bigger and elongated speckles. Bigger speckles have been reported under different cellular conditions, such as transcriptional inhibition, heat-shock, heavy metal stress, and during the transition from late G2 into prophase [22]. Interestingly, bigger speckles have also been detected when knocking down core components such as SON [15], further supporting RCOR2 as a core component. These morphological changes appeared to be post-translational, as we did not find significant changes in the protein and mRNA levels of speckle components, suggesting RCOR2 is regulating the dynamics of nuclear speckles rather than their total levels. Because nuclear speckle fusion has been recently reported [22], and bigger speckles could be the result of increased fusion, it is tempting to suggest that RCOR2-lacking cells could have altered fusion rates among speckles. We propose RCOR2 as one of the regulators of nuclear speckles morphology.

**Fig. 7:**
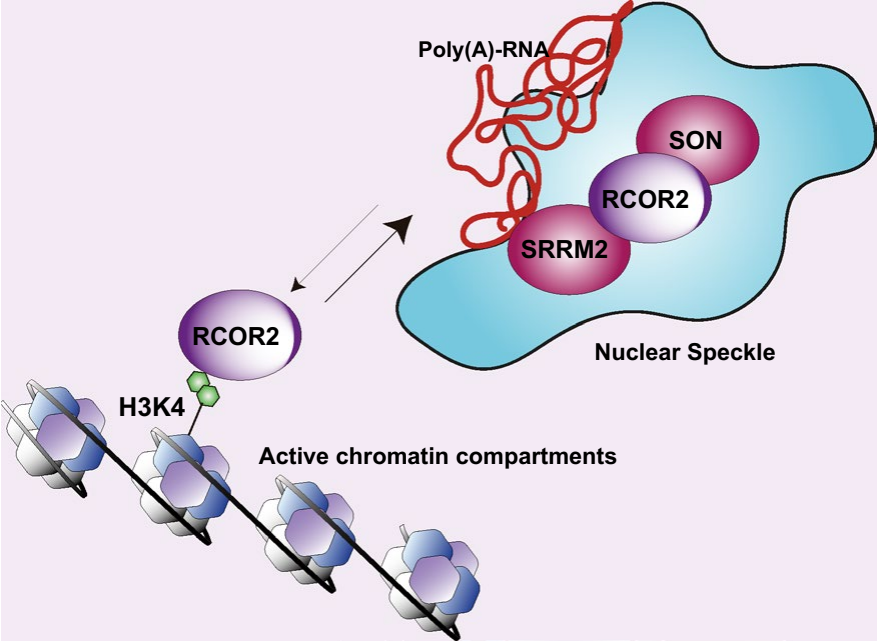
Working model. Our model illustrates both the nuclear speckle, and the chromatin-centric views of RCOR2 localization

As discussed above, we are also aware of the chromatin occupancy of RCOR2, which has been described near to the mark H3K4me1 [10], which is enriched in the two active chromatin subcompartments reported to date by high resolution Hi-C approaches [36]. These regions, enriched in highly expressed genes and super-enhancers, are commonly found in close proximity to nuclear speckles, and different reports have suggested that speckles can enhance transcription at these compartments [32, 37, 38]. Thus, our results could be explained recalling the two main RCOR2 locations, nuclear speckles and chromatin. From the chromatin-centric view, we know that chromatin environments limit the movements needed for nuclear speckle fusion events [22]. Thus, RCOR2 exerting transcriptional regulation at active compartments could impact their crosstalk with nuclear speckles and their morphology. Similarly, from a speckle-centric view, RCOR2 could be understood as a core factor that balances nuclear speckle fusion events. We suggest that both fractions might be functionally interacting to modulate nuclear speckle morphology (Fig. 7).

## Conclusions

Altogether, our study demonstrated the presence of RCOR2 at the core of nuclear speckles and its interaction with their core components. This novel observation is an RCOR2-specific feature, given that its homolog RCOR1 was not found at these territories. RCOR2 localization did not change upon different chemical treatments that compromised nuclear speckle morphology, but it was abolished under RNAse A treatment, suggesting RCOR2 interaction with RNAs is important for its intranuclear distribution. Finally, functional assays revealed RCOR2 as a protein that helps shaping the nuclear speckle morphology, favoring a circular and less-elongated conformation.

## Materials and methods

### Animals

One adult male C57BL/6 mouse was used for immunofluorescence tissue studies. This mouse came from the Center for Innovation Biomedical Experimental Models (CIBEM) of the Pontificia Universidad Catolica. This mouse was maintained in a four-colony on a 12-h-light/12-h-dark cycle with food and water available ad libitum. The procedures (protocol number 180808005) were conducted following national and institutional policies (Comisión Nacional de Investigación Científica y Tecnológica [CONICYT] and Pontificia Universidad Católica de Chile).

### Cell culture

HT22, HeLa, N2A, PC-12 and HEK293-T cells were cultured in Dulbecco’s modified Eagle’s medium (DMEM, Gibco) supplemented with 10% fetal bovine serum (FBS, Gibco) and 1% penicillin/streptomycin. PC-12 cell medium was supplemented with 5% horse serum. Cells grew at 37ºC in an atmosphere containing 5% CO_2_.

### Antibodies

Rabbit polyclonal anti-RCOR2 (Sigma HPA021638, lot number A117885), anti-coilin antibody (Genetex, GTX112570), anti-H3K9me3 (Abcam, ab8898), anti-HP1α (Cell Signaling, #2616S), anti-LSD1 (Abcam, ab17721), anti-SON (Thermo Fisher, #PA5-65107), anti-SRRM2 (Thermo Fisher, #PA5-66827), anti-HDAC2 (Abcam, #ab7029) and anti-GAPDH (Cell Signaling, #2118S). Rabbit monoclonal anti-HA (Cell Signaling, #3724S). We used anti-SC35 mouse monoclonal antibodies from (Genetex GTX11826) and (Sigma SAB4200725). Recently, it was shown that this monoclonal antibody recognizes the SRRM2 and SRSF7 proteins but does not recognize SC35 [14]. Mouse monoclonal anti-nucleophosmin (Abcam, ab10530), anti-SRSF1/ASF-1 (Millipore-Sigma, #MABE936). Alexa Fluor® 488 AffiniPure Fab Fragment Goat Anti-Rabbit IgG (H + L) (Jackson Immunoresearch, 111-547-003). AlexaFluor 594-conjugated donkey anti-rabbit IgG antibody (Invitrogen, Thermo Fisher, R37119). AlexaFluor 488-conjugated donkey anti-mouse IgG antibody (Invitrogen, Thermo Fisher, R37114).

### Cell immunofluorescence

Cells were fixed and permeabilized as previously shown [11]. For double labeling of RCOR2 and markers of nuclear bodies with antibodies raised in the same species, we performed anti-RCOR2 incubation first. Then we used a 10X excess (20 μg/mL) of monovalent Alexa Fluor 488 affinity-purified Fab fragment anti-Rabbit IgG (H + L) (Jackson Immunoresearch, 111-547-003), according to manufacturer instructions. After five consecutive 5 min 1X PBS washes, we incubated cells with the second primary antibody and second secondary antibody according to the regular protocol. For tissue immunofluorescence, we followed a protocol already described by our group [11].

### Poly(A)-RNA in situ hybridization

After immunostaining, cells were post-fixed in 4% paraformaldehyde-supplemented 1X PBS during 15 min at room temperature and washed 3 times in 1X PBS. Then cells were rinsed and dehydrated through 2-min sequential incubations in 70, 80, 90 and 100% V/V ethanol. We used a final concentration of 1 ng/μL of Cy5-labeled Oligo-dT_(50)_ probes in Hybes buffer (2X SSC pH 7.0, 10% m/V dextran sulphate, 25% V/V formamide, 100 ng/μL mouse cot-1 DNA). Probe was denatured at 80 °C for 5 min and then it was slowly cooled until reaching 37 °C. Cells were incubated with the probe 2 h at room temperature, followed by 3 washes in 4X SSC, 3 times in 2X SSC, DNA staining and mounting.

### Microscopy

Images were acquired on an Olympus DS-Fi2 epifluorescence microscope, using 40X and 100X Olympus UplanFI oil immersion objectives, a Nikon DS-fi2 camera and the Q-Imaging capture software. Confocal acquisition was carried out at Unidad de Microscopía Avanzada (UMA), Pontificia Universidad Católica de Chile. Cells were imaged on a Nikon Eclipse C2 Si Confocal Spectral Microscope with NIS-Elements C software. High-resolution confocal images were acquired at UMA and Massachusetts General Hospital (MGH) Program in Membrane Biology (PMB) Microscopy Core Facility, using the Zeiss LSM 800 Airyscan confocal microscope and Zeiss LSM 880 Airyscan confocal microscope, respectively, with Airyscan acquisition mode and conventional super-resolution processing using the ZEN software by Zeiss.

### Image analyses

Fiji was used to visualize images at first, which were further adjusted for contrast and/or brightness and assembled using Adobe Illustrator. Colocalization analyses were performed on ImageJ software (NIH, Baltimore, MD) by using the JACoP (Just another colocalization plugin) plugin [39] to determine thresholded Manders’ coefficients and Van Steensel parameters for single Z-stacks of images. Fluorescence intensity was measured using raw integrated densities of each cell over background measurements. Intensities were normalized as the percentage of total fluorescence counts. The spatial correlation of the fluorescence intensities of three-color images was performed on ImageJ by drawing a 10–15 μm line and then measuring the fluorescence intensity of each channel every 0.0155 μm. Elongation and circularity measurements were performed after segmenting representative images, identifying individual particles. All measurements were computed after binarizing microscopy images and applying edge detection to identify speckles of interest. A minimum threshold value of 300 pixels was used to avoid noise. Circularity is defined as 2π *r*/*p*, where *p* is the polygonal length and *r* equivalent disk radius. A perfect circle will have a value of 1, whereas non-circular objects will approach 0. Elongation is computed as ratio of 1- width/height.

### Immunoprecipitation

Cells were lysed in Immunoprecipitation buffer (50 mM Tris–HCl pH 7.5, 150 mM NaCl, 0.5% Sodium deoxycholate, 1% NP40 and protease inhibitors. Sonication was applied to solubilize chromatin-bound material. Binding and elution reactions were performed as previously described [4].

### RNA immunoprecipitation (RIP)

Nuclear HT22 pellets were prepared and incubated in RIP-Nuclear Lysis buffer (1 × PBS pH 7.7, 1% NP40, 0.5% sodium deoxycholate, 100 U/mL Superase In RNase Inhibitor (Thermo-Fisher Scientific) and 1 × protease inhibitor cocktail (Roche)) for 30 min with rotation at 4 °C. RIP was performed as previously described [40]. Bound RNAs were recovered with Trizol LS (Thermo-Fisher Scientific) and purified using Direct-zol RNA Miniprep Plus Kit (Zymo Research). MALAT1, 7SK and HOTAIR RNAs were detected by random primers-mediated reverse transcription followed by qPCR using the primer pairs: MALAT1 Forward: GCATGCCAGTGTGCAAGAAA, Reverse: ACCCGCAAAGGCCTACATAC. 7SK Forward: CCCTGCTAGAACCTCCAAAC, Reverse: TGGAGTCTTGGAAGCTTGACT. HOTAIR Forward: CCAGTGGCAGGATAGGCACA, Reverse: CGTGGTCAGATCGCTGGTCA.

### Subcellular fractionation and sequential extraction of chromatin-bound proteins

Cytosolic extracts were prepared as previously described [41]. Nuclei were collected and washed 3 times in hypotonic buffer, then were sequentially resuspended in nuclear extraction buffers (20 mM Tris, pH 7.9, 25% glycerol, 1.5 mM MgCl2, 0.2 mM EDTA, 0.5 mM DTT, 0.5 mM PMSF and 1X protease inhibitor complex (Roche)), starting with 100 mM NaCl and increasing salt concentration by steps of 100 mM until 600 mM NaCl was reached. For each step, nuclei were incubated for 7 min at 4 °C and then centrifuged at 4000×*g* for 5 additional minutes. Supernatants were collected and cleared by centrifugation at 15,000×*g* for 30 min at 4 °C. Final pellet was solubilized directly on 2X Laemmli buffer.

### RNA interference

Hela cells were seeded at 80% confluency and 6 h later were transfected with 100 nM siGenome Human RCOR2 siRNA (Horizon Discovery Biosciences, #M-018296-00-0005) or non targeting siRNA pool (Horizon Discovery Biosciences, #D-001810-10-05) using Lipofectamine RNAiMAX transfection reagent (Thermo Fisher, #13778075) and Opti-MEM reduced serum medium supplemented with GlutaMAX (Thermo Fisher, #51985034) according to manufacturer instructions. Cells were harvested 24 h after transfection and used for RT-qPCR, Western blot and or immunostaining. RT-qPCR was performed using the following primer pairs: RCOR2-A forward: TACAACATTGAGCAGGCGCT, reverse: TGGATCCGCTGGAAGCATTT. RCOR2-B forward: GCTTCTGTGGCATAAGCACG, reverse: TCAACTTGTCAGGCAGCATC. SRRM1 forward: ATCTCGTTCACGGTCACCAC, reverse: CCTGGAGACACAGAAACACGA. SRRM2 forward: CCACAGAGACGGAGCTGTTT, reverse: GTTGTTCTCGACGTCACCCT. SON Forward:GAGCCCCCAGTAGCAAAAGT, reverse: CCGATGGTACGTCTACAGGC. GAPDH forward: ATGAATACGGCTACAGCAACAGG, reverse: CTCTTGCTCAGTGTCCTTGCTG.

### RCOR2 overexpression

HeLa cells were transfected using pcDNA3.1 or pcDNA3.1-HA-RCOR2 (insert: NM_054048.4) using a ratio of 500 ng Plasmid DNA every 1 uL Lipofectamine 3000 transfection reagent (Thermo Fisher, #L3000075) and Opti-MEM reduced serum medium supplemented with GlutaMAX (Thermo Fisher, #51985034) according to manufacturer instructions. Cells were harvested 24 h after transfection and used for RT-qPCR, Western blot and or immunostaining. PC12 cells were transfected using pcDNA3.1-Myc-RCOR2 (insert: NM_173587.4) using a ratio of 500 ng Plasmid DNA every 1 uL Lipofectamine 3000 transfection reagent (Thermo Fisher, #L3000075) and Opti-MEM reduced serum medium supplemented with GlutaMAX (Thermo Fisher, #51985034) according to manufacturer instructions.

## Supplementary Information

**Additional file 1: Figure S1.** Anti-RCOR2 antibody validation. (A) Western blot of total extracts from N2A, HEK293T and PC12 cells using the anti-RCOR2 antibody. (B) HEK293T cells were transfected to ectopically overexpress RCOR2-Myc. Western blot was performed using the anti-RCOR2 antibody. β-Actin was assayed as a loading control. (C) Confocal image showing immunostaining of anti Myc-epitope (red) and anti-RCOR2 (green) on transiently transfected PC12 cells with RCOR2-Myc. (D) Peptide competition assay. RCOR2 immunostaining was performed on HT22 cells. Anti-RCOR2 antibody was pre-incubated with RCOR2 (2–61) peptide or H3 (1–20) peptide (negative control) at 1:1 and 1:5 antibody:peptide molar ratios, respectively. (E) Western blot analysis of RCOR2 after HEK293T cells were transfected with siRNA targeting RCOR2. β-Tubulin was assayed as loading control. **(F)** RCOR2 immunofluorescence was performed on HT22 cells that were transduced with lentiviral particles to perform shRNA mediated knockdown of RCOR2. Fluorescence intensity was quantitated on the right plot.

**Additional file 2: Figure S2. **(A) Tissue immunofluorescence of SRRM2 (green) and RCOR2 (red) in prefrontal cortex slices. The right panels show a zoomed-in nucleus, illustrating speckles found in single nuclei of the brain tissues. (B) Biochemical fractionation of HT22 cells by sequential salt extraction of nuclear proteins. The left schematic workflow indicates the overall procedure, showing how cytosolic (S1), nuclear (P1), and chromatin fractions (P12) were obtained. For sequential nuclear extractions, nuclear extracts at different salt concentrations are labeled as N100, where N means nuclear extract and 100 means 100 mM NaCl. (C) Western blot showing the co-immunoprecipitation of SRSF7 with RCOR2 and LSD1 using HEK293T cells as input. (D) Confocal images of RCOR2 (green), SRRM2 (red), and Poly(A)-RNA (magenta) triple staining in permeabilized, mock-treated HT22 cells before fixation. The merged image includes Hoechst DNA staining.

**Additional file 3: Figure S3.** (A, B) HeLa cells were stained against RCOR2 (green) and SRRM2 (red) after treatments with high doses of actinomycin D (A) and isoginkgetin (D). Images are representative of two independent experiments.

**Additional file 4: Figure S4.** (A, B) Segmented images extracted from SRRM2 immunostaining in conditions where RCOR2 was knocked down (A) or overexpressed (B). Colored gradient shows relative values according to the particle sizes.

**Additional file 5: Video S1.** RCOR2 is recruited to nuclear speckles in the mouse brain. Z-stack visualization of tissue immunofluorescence of SRRM2 (red) and RCOR2 (green) in mice striatum slices.

## Data Availability

Not applicable.

## References

[1] Millard CJ, Watson PJ, Fairall L, Schwabe JW (2013). An evolving understanding of nuclear receptor coregulator proteins. J Mol Endocrinol.

[2] Ballas N, Grunseich C, Lu DD, Speh JC, Mandel G (2005). REST and its corepressors mediate plasticity of neuronal gene chromatin throughout neurogenesis. Cell.

[3] Shi YJ, Matson C, Lan F, Iwase S, Baba T, Shi Y (2005). Regulation of LSD1 histone demethylase activity by its associated factors. Mol Cell.

[4] Barrios AP, Gomez AV, Saez JE, Ciossani G, Toffolo E, Battaglioli E, Mattevi A, Andres ME (2014). Differential properties of transcriptional complexes formed by the CoREST family. Mol Cell Biol.

[5] Song Y, Dagil L, Fairall L, Robertson N, Wu M, Ragan TJ, Savva CG, Saleh A, Morone N, Kunze MBA (2020). Mechanism of crosstalk between the LSD1 demethylase and HDAC1 deacetylase in the CoREST complex. Cell Rep.

[6] Pilotto S, Speranzini V, Tortorici M, Durand D, Fish A, Valente S, Forneris F, Mai A, Sixma TK, Vachette P (2015). Interplay among nucleosomal DNA, histone tails, and corepressor CoREST underlies LSD1-mediated H3 demethylation. Proc Natl Acad Sci U S A.

[7] Upadhyay G, Chowdhury AH, Vaidyanathan B, Kim D, Saleque S (2014). Antagonistic actions of Rcor proteins regulate LSD1 activity and cellular differentiation. Proc Natl Acad Sci USA.

[8] Yang P, Wang Y, Chen J, Li H, Kang L, Zhang Y, Chen S, Zhu B, Gao S (2011). RCOR2 is a subunit of the LSD1 complex that regulates ESC property and substitutes for SOX2 in reprogramming somatic cells to pluripotency. Stem Cells.

[9] Monaghan CE, Nechiporuk T, Jeng S, McWeeney SK, Wang J, Rosenfeld MG, Mandel G (2017). REST corepressors RCOR1 and RCOR2 and the repressor INSM1 regulate the proliferation-differentiation balance in the developing brain. Proc Natl Acad Sci USA.

[10] Wang Y, Wu Q, Yang P, Wang C, Liu J, Ding W, Liu W, Bai Y, Yang Y, Wang H (2016). LSD1 co-repressor Rcor2 orchestrates neurogenesis in the developing mouse brain. Nat Commun.

[11] Saez JE, Gomez AV, Barrios AP, Parada GE, Galdames L, Gonzalez M, Andres ME (2015). Decreased expression of CoREST1 and CoREST2 together with LSD1 and HDAC1/2 during neuronal differentiation. PLoS ONE.

[12] Thiry M (1995). The interchromatin granules. Histol Histopathol.

[13] Zhu L, Brangwynne CP (2015). Nuclear bodies: the emerging biophysics of nucleoplasmic phases. Curr Opin Cell Biol.

[14] Ilik IA, Malszycki M, Lubke AK, Schade C, Meierhofer D, Aktas T (2020). SON and SRRM2 are essential for nuclear speckle formation. Elife.

[15] Fei J, Jadaliha M, Harmon TS, Li ITS, Hua B, Hao Q, Holehouse AS, Reyer M, Sun Q, Freier SM (2017). Quantitative analysis of multilayer organization of proteins and RNA in nuclear speckles at super resolution. J Cell Sci.

[16] Kolossov VL, Sivaguru M, Huff J, Luby K, Kanakaraju K, Gaskins HR (2018). Airyscan super-resolution microscopy of mitochondrial morphology and dynamics in living tumor cells. Microsc Res Tech.

[17] Girard C, Will CL, Peng J, Makarov EM, Kastner B, Lemm I, Urlaub H, Hartmuth K, Luhrmann R (2012). Post-transcriptional spliceosomes are retained in nuclear speckles until splicing completion. Nat Commun.

[18] Daguenet E, Baguet A, Degot S, Schmidt U, Alpy F, Wendling C, Spiegelhalter C, Kessler P, Rio MC, Le Hir H (2012). Perispeckles are major assembly sites for the exon junction core complex. Mol Biol Cell.

[19] Hall LL, Smith KP, Byron M, Lawrence JB (2006). Molecular anatomy of a speckle. Anat Rec A Discov Mol Cell Evol Biol.

[20] Sytnikova YA, Kubarenko AV, Schafer A, Weber AN, Niehrs C (2011). Gadd45a is an RNA binding protein and is localized in nuclear speckles. PLoS ONE.

[21] Becerra S, Montes M, Hernandez-Munain C, Sune C (2015). Prp40 pre-mRNA processing factor 40 homolog B (PRPF40B) associates with SF1 and U2AF65 and modulates alternative pre-mRNA splicing in vivo. RNA.

[22] Kim J, Han KY, Khanna N, Ha T, Belmont AS (2019). Nuclear speckle fusion via long-range directional motion regulates speckle morphology after transcriptional inhibition. J Cell Sci.

[23] O'Brien K, Matlin AJ, Lowell AM, Moore MJ (2008). The biflavonoid isoginkgetin is a general inhibitor of Pre-mRNA splicing. J Biol Chem.

[24] Hochberg-Laufer H, Schwed-Gross A, Neugebauer KM, Shav-Tal Y (2019). Uncoupling of nucleo-cytoplasmic RNA export and localization during stress. Nucleic Acids Res.

[25] Kurogi Y, Matsuo Y, Mihara Y, Yagi H, Shigaki-Miyamoto K, Toyota S, Azuma Y, Igarashi M, Tani T (2014). Identification of a chemical inhibitor for nuclear speckle formation: implications for the function of nuclear speckles in regulation of alternative pre-mRNA splicing. Biochem Biophys Res Commun.

[26] Salichs E, Ledda A, Mularoni L, Alba MM, de la Luna S (2009). Genome-wide analysis of histidine repeats reveals their role in the localization of human proteins to the nuclear speckles compartment. PLoS Genet.

[27] Wu X, Li H, Park EJ, Chen JD (2001). SMRTE inhibits MEF2C transcriptional activation by targeting HDAC4 and 5 to nuclear domains. J Biol Chem.

[28] Alexander KA, Cote A, Nguyen SC, Zhang L, Gholamalamdari O, Agudelo-Garcia P, Lin-Shiao E, Tanim KMA, Lim J, Biddle N (2021). p53 mediates target gene association with nuclear speckles for amplified RNA expression. Mol Cell.

[29] Kruhlak MJ, Lever MA, Fischle W, Verdin E, Bazett-Jones DP, Hendzel MJ (2000). Reduced mobility of the alternate splicing factor (ASF) through the nucleoplasm and steady state speckle compartments. J Cell Biol.

[30] Phair RD, Misteli T (2000). High mobility of proteins in the mammalian cell nucleus. Nature.

[31] Hochberg-Laufer H, Neufeld N, Brody Y, Nadav-Eliyahu S, Ben-Yishay R, Shav-Tal Y (2019). Availability of splicing factors in the nucleoplasm can regulate the release of mRNA from the gene after transcription. PLoS Genet.

[32] Quinodoz SA, Ollikainen N, Tabak B, Palla A, Schmidt JM, Detmar E, Lai MM, Shishkin AA, Bhat P, Takei Y (2018). Higher-order inter-chromosomal hubs shape 3D genome organization in the nucleus. Cell.

[33] Steurer B, Janssens RC, Geverts B, Geijer ME, Wienholz F, Theil AF, Chang J, Dealy S, Pothof J, van Cappellen WA (2018). Live-cell analysis of endogenous GFP-RPB1 uncovers rapid turnover of initiating and promoter-paused RNA Polymerase II. Proc Natl Acad Sci USA.

[34] Spector DL, Fu XD, Maniatis T (1991). Associations between distinct pre-mRNA splicing components and the cell nucleus. EMBO J.

[35] Bernard D, Prasanth KV, Tripathi V, Colasse S, Nakamura T, Xuan Z, Zhang MQ, Sedel F, Jourdren L, Coulpier F (2010). A long nuclear-retained non-coding RNA regulates synaptogenesis by modulating gene expression. EMBO J.

[36] Rao SS, Huntley MH, Durand NC, Stamenova EK, Bochkov ID, Robinson JT, Sanborn AL, Machol I, Omer AD, Lander ES (2014). A 3D map of the human genome at kilobase resolution reveals principles of chromatin looping. Cell.

[37] Chen Y, Zhang Y, Wang Y, Zhang L, Brinkman EK, Adam SA, Goldman R, van Steensel B, Ma J, Belmont AS (2018). Mapping 3D genome organization relative to nuclear compartments using TSA-Seq as a cytological ruler. J Cell Biol.

[38] Kim J, Venkata NC, Hernandez Gonzalez GA, Khanna N, Belmont AS (2020). Gene expression amplification by nuclear speckle association. J Cell Biol.

[39] Bolte S, Cordelieres FP (2006). A guided tour into subcellular colocalization analysis in light microscopy. J Microsc.

[40] Jeon Y, Lee JT (2011). YY1 tethers Xist RNA to the inactive X nucleation center. Cell.

[41] Saavedra F, Marty-Lombardi S, Loyola A (2018). Characterization of posttranslational modifications on histone variants. Methods Mol Biol.

