# Supplementary figures and images for: Revealing RCOR2 as a regulatory component of nuclear speckles
Source: Epigenetics Chromatin. 2021 Nov 24;14:51. doi: 10.1186/s13072-021-00425-4 (PMC8611983; doi:10.1186/s13072-021-00425-4)

Figure S1. Anti-RCOR2 Antibody Validation

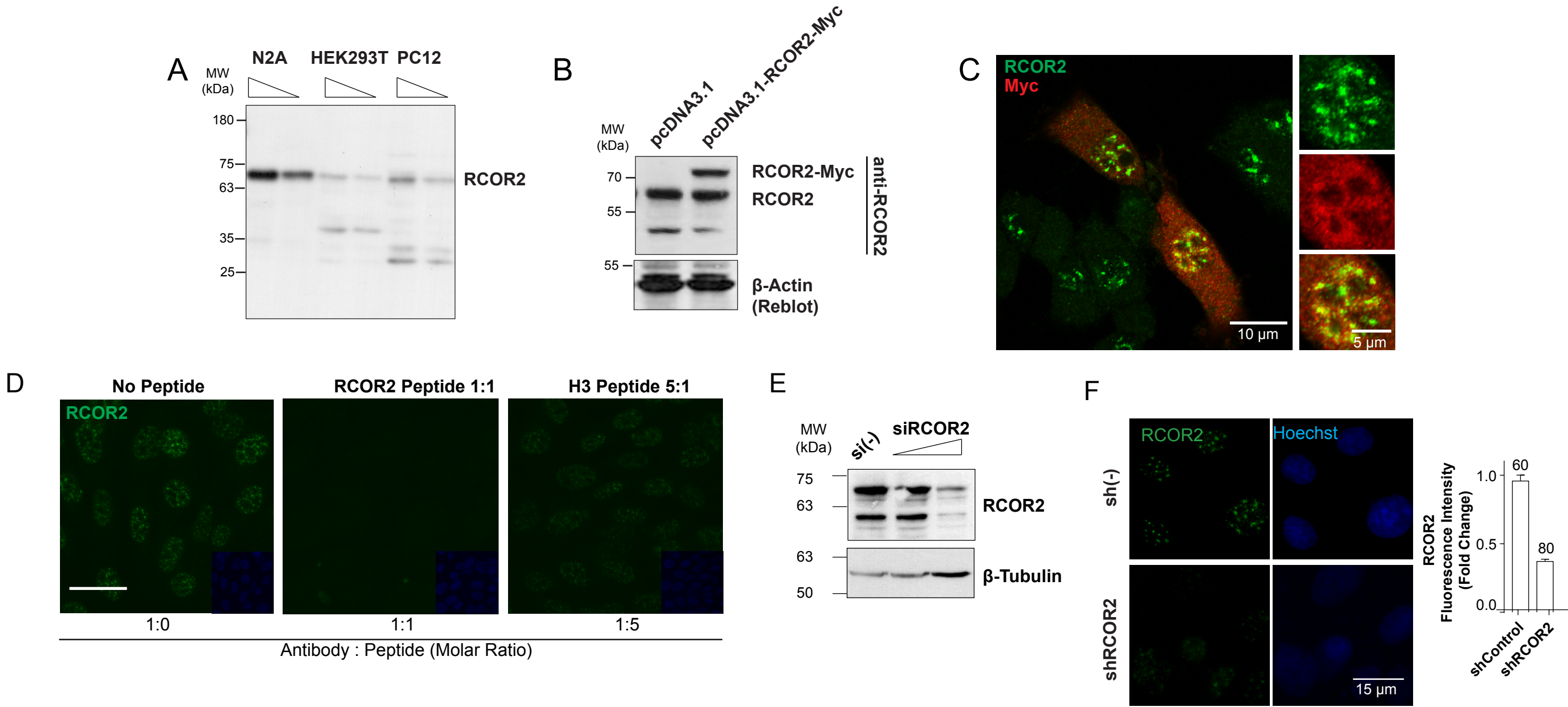

Supplement: Supplementary file 1 — Additional file 1: Figure S1. Anti-RCOR2 antibody validation. (A) Western blot of total extracts from N2A, HEK293T and PC12 cells using the anti-RCOR2 antibody. (B) HEK293T cells were transfected to ectopically overexpress RCOR2-Myc. Western blot was performed using the anti-RCOR2 antibody. β-Actin was assayed as a loading control. (C) Confocal image showing immunostaining of anti Myc-epitope (red) and anti-RCOR2 (green) on transiently transfected PC12 cells with RCOR2-Myc. (D) Peptide competition assay. RCOR2 immunostaining was performed on HT22 cells. Anti-RCOR2 antibody was pre-incubated with RCOR2 (2–61) peptide or H3 (1–20) peptide (negative control) at 1:1 and 1:5 antibody:peptide molar ratios, respectively. (E) Western blot analysis of RCOR2 after HEK293T cells were transfected with siRNA targeting RCOR2. β-Tubulin was assayed as loading control. (F) RCOR2 immunofluorescence was performed on HT22 cells that were transduced with lentiviral particles to perform shRNA mediated knockdown of RCOR2. Fluorescence intensity was quantitated on the right plot. [file 13072_2021_425_MOESM1_ESM.pdf]

Figure S2.

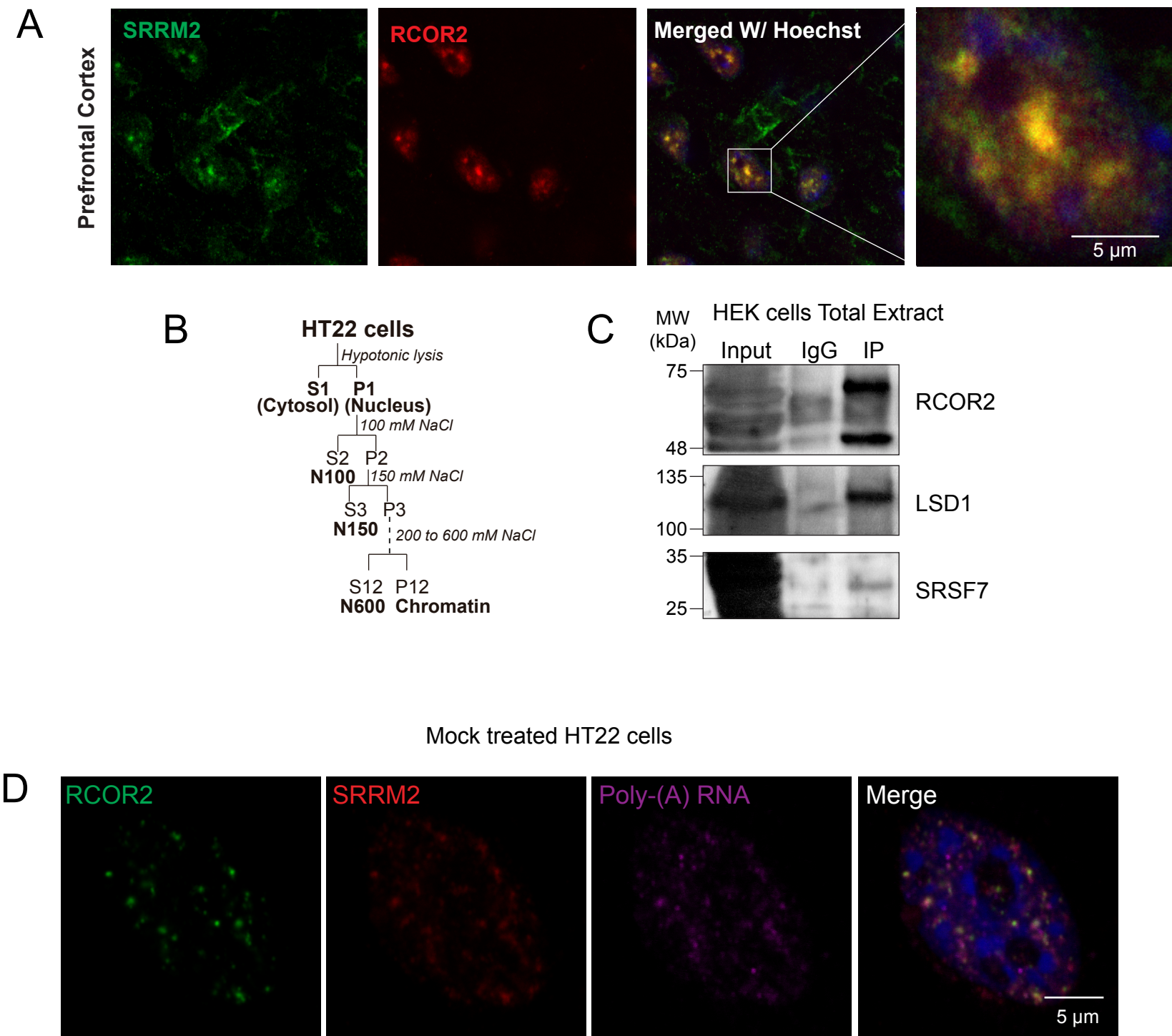

Supplement: Supplementary file 2 — Additional file 2: Figure S2. (A) Tissue immunofluorescence of SRRM2 (green) and RCOR2 (red) in prefrontal cortex slices. The right panels show a zoomed-in nucleus, illustrating speckles found in single nuclei of the brain tissues. (B) Biochemical fractionation of HT22 cells by sequential salt extraction of nuclear proteins. The left schematic workflow indicates the overall procedure, showing how cytosolic (S1), nuclear (P1), and chromatin fractions (P12) were obtained. For sequential nuclear extractions, nuclear extracts at different salt concentrations are labeled as N100, where N means nuclear extract and 100 means 100 mM NaCl. (C) Western blot showing the co-immunoprecipitation of SRSF7 with RCOR2 and LSD1 using HEK293T cells as input. (D) Confocal images of RCOR2 (green), SRRM2 (red), and Poly(A)-RNA (magenta) triple staining in permeabilized, mock-treated HT22 cells before fixation. The merged image includes Hoechst DNA staining. [file 13072_2021_425_MOESM2_ESM.pdf]

**Figure S3.**

**A**

100 nM Actinomycin D

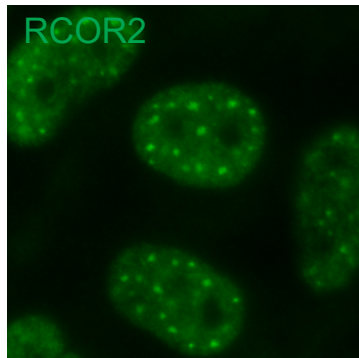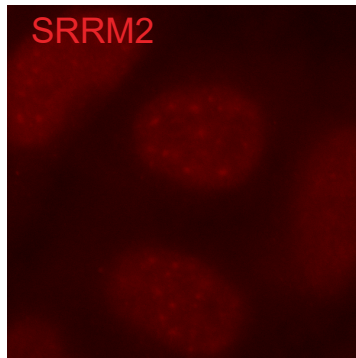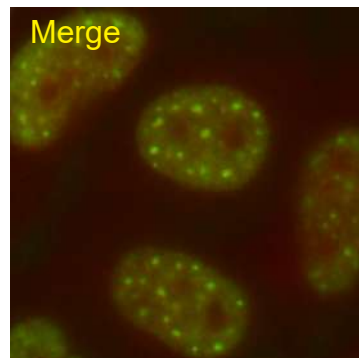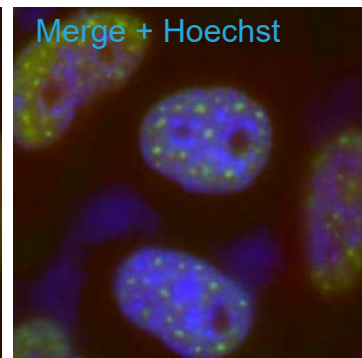

**B**

100  $\mu$ M Isoginkgetin

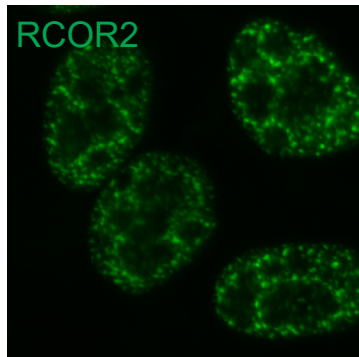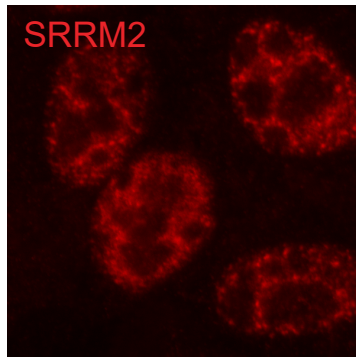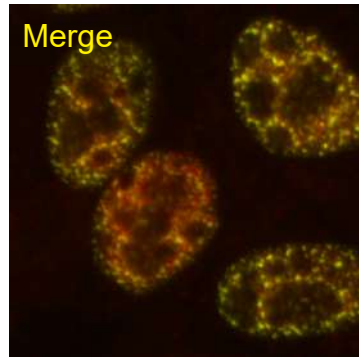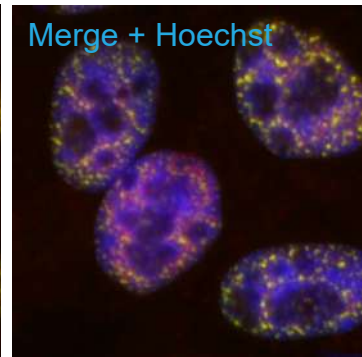

Supplement: Supplementary file 3 — Additional file 3: Figure S3. (A, B) HeLa cells were stained against RCOR2 (green) and SRRM2 (red) after treatments with high doses of actinomycin D (A) and isoginkgetin (D). Images are representative of two independent experiments. [file 13072_2021_425_MOESM3_ESM.pdf]

# Figure S4.

A

siRNA Control

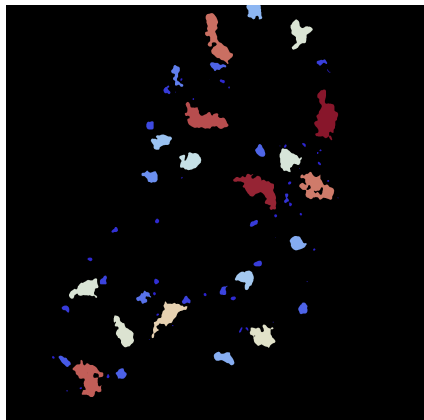

siRNA RCOR2

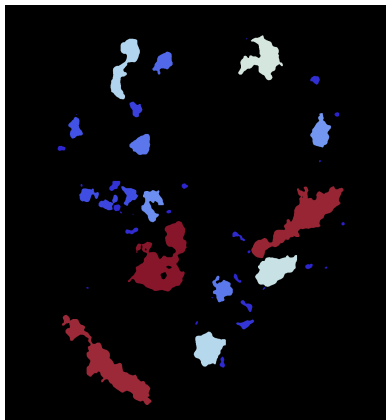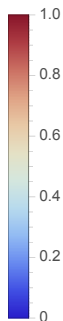

B

pcDNA3.1

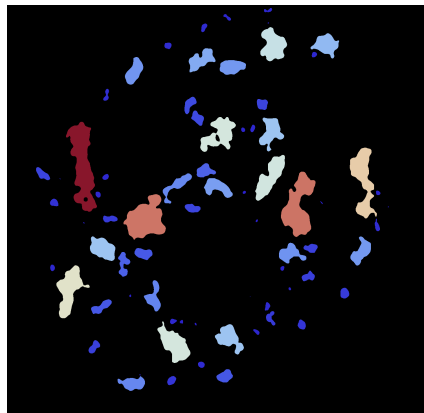

pcDNA3.1-HA-RCOR2

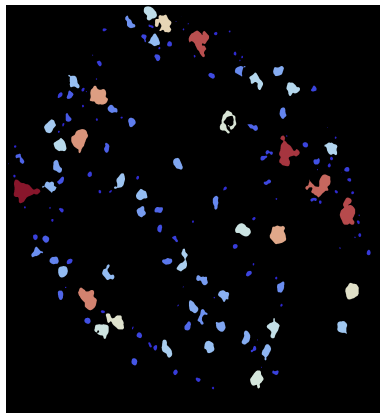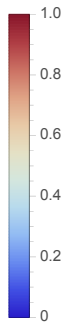

Supplement: Supplementary file 4 — Additional file 4: Figure S4. (A, B) Segmented images extracted from SRRM2 immunostaining in conditions where RCOR2 was knocked down (A) or overexpressed (B). Colored gradient shows relative values according to the particle sizes. [file 13072_2021_425_MOESM4_ESM.pdf]
